# Widespread elevated iridium in Upper Triassic–Lower Jurassic strata of the Newark Supergroup: implications for use as an extinction marker

**DOI:** 10.1038/s41598-020-76238-4

**Published:** 2020-11-11

**Authors:** Lawrence H. Tanner, Frank T. Kyte, John H. Puffer

**Affiliations:** 1 Department of Biological and Environmental Sciences, Le Moyne College, Syracuse, NY 13214 USA; 2 Institute for Geophysics and Planetary Physics, University of California, Los Angeles, CA 90095-1567 USA; 3 Department of Earth and Environmental Science, Rutgers University, Newark, NJ 07102 USA

**Keywords:** Solid Earth sciences, Geochemistry, Volcanology

## Abstract

Anomalous levels of iridium in sedimentary strata are associated with mass extinction events caused by impact events. In the case of the end-Triassic extinction event, the anomalies as well as the extinctions are linked to the eruption of the Central Atlantic Magmatic Province (CAMP) flood basalts. We report new data on concentrations of iridium in continental strata of the Fundy, Deerfield, Hartford and Newark basins, both above and below the oldest CAMP flows in these basins, that demonstrate that these anomalies are more common than previously known. We conclude that the enrichments were at least in some instances likely derived locally by concentration due to leaching directly from the lavas into sediments proximal to the CAMP flows due to post-eruptive hydrothermal activity. In other instances, the enrichments likely record the global fallout of aerosols and/or ash particles during the eruptions of the CAMP basalts. The common association of the highest levels of enrichment with organic matter suggests either redox control or stabilization by formation of organometallic complexes following post-eruptive redistribution. These findings demonstrate the importance of considering the distribution and magnitude of iridium anomalies in considering the source of the iridium and possible extinction mechanisms.

## Introduction

The occurrence of iridium (Ir) in sediments surrounding paleontological event boundaries has long garnered interest for its potential in identifying bolide impact as a possible driver of mass extinction. This interest has extended to the biodiversity crisis at the Triassic–Jurassic boundary (TJB). This boundary has long been identified as one of the “big five” mass extinctions of the Phanerozoic^1^. More precisely, the Late Triassic was an extended interval of high biotic turnover rates punctuated by discrete extinction events, the last of which occurred during the latest Rhaetian, an event often termed the end-Triassic extinction (ETE)^2–5^. In recent years, the focus of attention in the study of the end-Triassic extinctions has been the environmental effects of the eruption of the flood basalts of the Central Atlantic Magmatic Province (CAMP). It is now well-established that the products of the CAMP eruptions span the system boundary^6–9^, with the majority of the total lava volume ejected during an initial eruptive episode prior to the paleontologically-defined TJB^10,11^. Within the basins of the Newark Supergroup, the eruptions proceeded in three main episodes, separated by eruptive hiatuses during which sediments accumulated, across a total interval of ca. 700 ky. Notably, several studies have suggested that the eruptive sequence initiated slightly earlier in Morocco than in North America^12,13^. Because much of the area originally covered by lavas has been deeply eroded^14^, the original volume of the eruptive products is necessarily speculative. Most estimates are in the range of 2 to 6 × 10^6^ km^4,11^.

The ETE is now generally acknowledged as the consequence of outgassing during CAMP activity that caused multiple environmental disturbances through episodes of SO_2_-aerosol driven cooling and reduced primary productivity, CO_2_-forced radiative warming, volcanogenic heavy-metal toxicity and acidification of environments^4,5,11,15–19^. Estimation of the mass of outgassed CO_2_ and SO_2_ has proved a contentious issue given the uncertainties in the erupted volume, but most calculations have suggested the release of both CO_2_ and SO_2_ at the scale of 10^3^–10^4^ Gt^5,11^. The environmental effects of the release of these gases necessarily depends on the timing and rate of their release, however. A pronounced negative carbon-isotope excursion (CIE) that records a disturbance of the global carbon cycle has long been associated with the ETE in the marine realm^20–26^ and is now identified from terrestrial TJB sections^16,27^. In some sections this CIE corresponds to anomalous levels of Hg, considered a marker for volcanic activity^28,29^, lending support to the association of CAMP eruptions and the extinctions.

**Figure 1:**
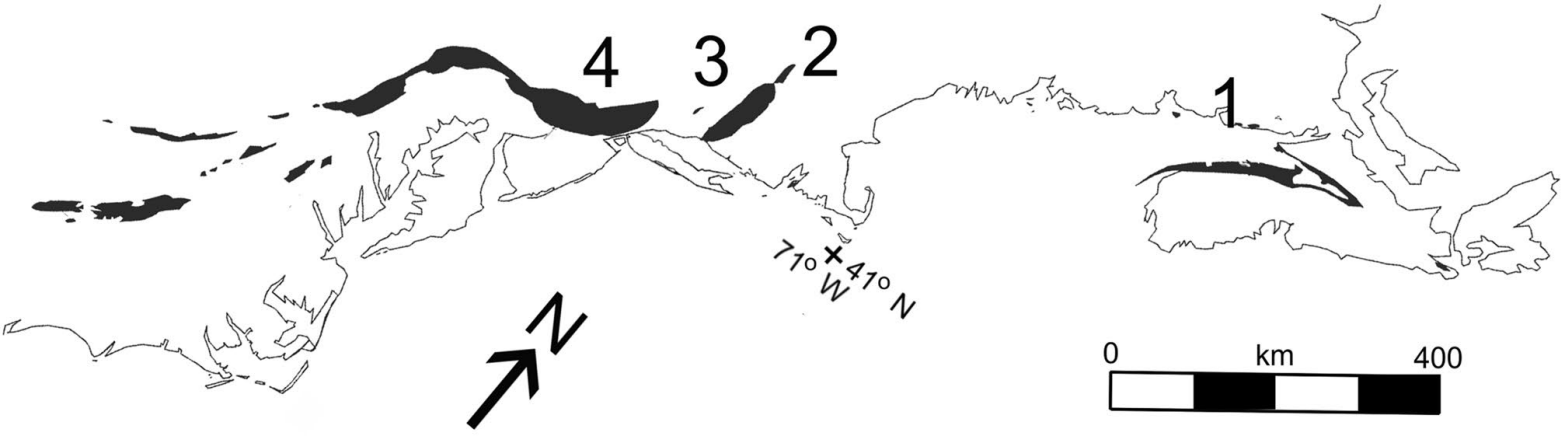
Map of Newark Supergroup basins. Simplified map of the eastern margin of North America showing location of the onshore rift basins of the Newark supergroup (shaded). Basins sampled in this study are: (1) Fundy; (2) Deerfield; (3) Hartford; (4) Newark.

To date, all reports of elevated Ir at the TJB in terrestrial sediments are from studies of the Newark Supergroup basins. The Newark Supergroup comprises the fill of the rift basins that formed along the eastern margin of North America during the Middle Triassic through Early Jurassic due to the rifting of Pangea (Fig. 1). The basins filled with mostly red-bed fluvial-lacustrine sediments and CAMP volcanics. They extend from the Canadian Maritimes to South Carolina, but erosion has removed the younger sedimentary formations and volcanics from the southern basins^14^.

**Figure 2:**
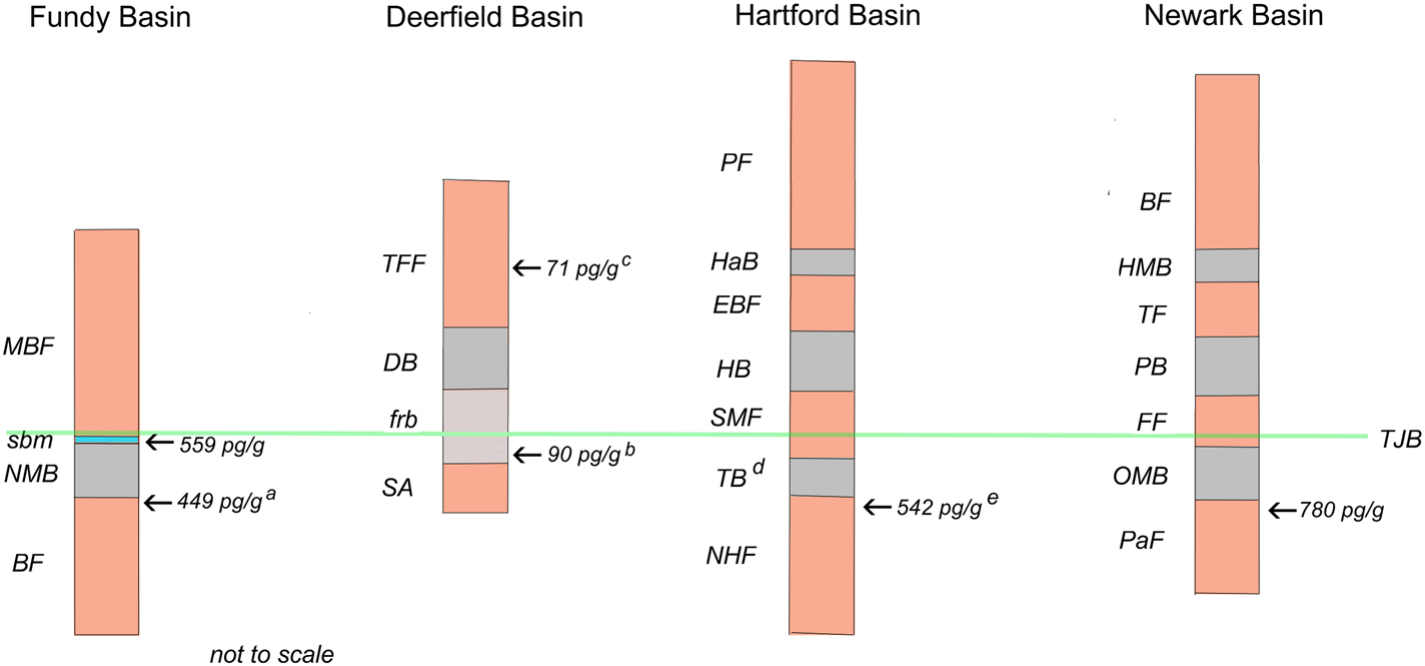
Stratigraphy of the studied basins. Northern basins of the Newark supergroup with peak Ir concentrations. BF, Blomidon Formation; NMB, North Mountain Basalt; sbm, Scots Bay Member of McCoy Brook Formation; MBF, McCoy Brook Formation; SA, Sugarloaf Arkose; frb, Fall River Beds; DB, Deerfield Basalt; TFF, Turners Falls Formation; NHF, New Haven Formation; TB, Talcott Basalt; SMF, Shuttle Meadow Formation; HB, Holyoke Basalt; EBF, East Berlin Formation; HaB, Hampden Basalt; PF, Portland Formation; PaF, Passaic Formation; OMB, Orange Mountain Basalt; FF, Feltville Formation; PB, Preakness Basalt; TF, Towaco Formation; HMB, Hook Mountain Basalt; BF, Boonton Formation; TJB, Triassic-Jurassic Boundary. ^a^data from^36^, ^b^sample from 3.0 m below DB is tentatively correlated with oldest CAMP volcanic units, ^c^sample from 1.5 m above DB is tentatively correlated with uppermost CAMP volcanic units, ^d^Talcott Basalt is not present in northern Hartford Basin, ^e^Holyoke *Clathropteris* locality is correlated as upper New Haven Formation.

Elevated Ir in TJB sediments was first described in the uppermost Passaic Formation (Upper Triassic), which underlies the Orange Mountain Basalt (OMB), the basal CAMP unit (Fig. 2), in the Jacksonwald syncline of the western Newark basin^30,31^. Measurements from four closely spaced outcrops spread over a total distance of ca. 1 km demonstrated a locally correlative horizon at which Ir was elevated to a maximum of 285 pg/g. No other platinum group elements (PGEs) were analyzed in this study, however. The horizon of Ir concentration was generally a carbonaceous shale with abundant trilete spores (the so-called “fern-spike”)^30,31^ that coincided with a palynological turnover then interpreted as the TJB. Despite the lack of supporting evidence, the authors considered the likelihood that this horizon recorded an extinction-driving bolide impact, although they conceded the possibility that instead it was related to the volcanic activity of CAMP. In more recent years, the impact hypothesis has generally been abandoned due to the lack of identification of shock-metamorphosed minerals, impact-melt spherules, an impact structure of appropriate age or other supporting evidence^32^. Moreover, there is now geochemical and sedimentary evidence from marine sections that CAMP volcanism coincided with the ETE^33,34^. Subsequent to the earlier work^30,31^, elevated Ir was identified at multiple horizons below the North Mountain Basalt (NMB) in the uppermost Blomidon Formation (Upper Triassic) in the Fundy basin (Fig. 2)^35,36^, potentially correlative with the horizon in the Passaic Formation^30,31^. These subsequent studies largely discounted the possibility of an impact origin and focused on derivation from the CAMP volcanics, either through aerosol deposition of PGEs from plumes during the eruptions, or remobilization from the overlying basalt by post-eruptive hydrothermal activity^37^.

Additional studies have identified enrichment of Ir in marine environments at or near the TJB, including deep-marine chert and shale-chert sections in Japan^38,39^ and shallower carbonate-siliciclastic sediments from the intracratonic Eiberg basin in the Northern Calcareous Alps^40^. All of these studies concluded that the elevation of Ir and associated PGEs in marine environments was potentially derived from atmospheric deposition of eruptive materials. No other Ir enrichments have been reported from terrestrial environments, however. For example, a terrestrial section in Poland was found to have no evidence of elevated Ir across the TJB^41^. In this study, we investigate the stratigraphic distribution and continuity of elevated PGEs in multiple basins of the Newark Supergroup and discuss the implications of the new data on possible modes of Ir concentration in the sedimentary formations surrounding the TJB.

## Results

### Fundy basin

Elevated Ir and associated PGEs were reported previously below the NMB in the uppermost strata of the Blomidon Formation^35,36^. In this study we report elevated Ir, Pd and Pt in strata overlying the NMB. The McCoy Brook Formation encompasses ca. 230 m of mostly red-bed strata directly above the NMB^42^. Locally, the Scots Bay Member, comprising up to nine meters of variegated mudstones and sandstones, onlaps the underlying NMB, forming the basal unit of the McCoy Brook Formation (Fig. 2). The basal centimeter contains a lag of basalt debris and the presence of abundant fish scales and bones demonstrates a lacustrine environment of deposition^42,43^. The presence of a Late Triassic pollen assemblage in the lowest few meters of the Scots Bay Member proves that the TJB occurs above the NMB^8^. We sampled the lowest 30 m of the McCoy Brook Formation, including the Scots Bay Member at two locations on the north shore of the Minas basin (see Supplemental information for locations). Samples from a ca. 0.1 m interval of purple mudstone at the base of the Scots Bay Member (latest Triassic in age) yielded the highest concentrations of Ir, up to 559 pg/g, and Pd and Pt concentrations of 2.4 ng/g and 2.9 ng/g, respectively (Table 1 and Supplemental Table 1a). Ir levels decrease by almost an order of magnitude within the next few decimeters of Scots Bay lacustrine strata and remain low in thinly laminated lacustrine mudstones higher in the McCoy Brook Formation (at about 30 m above the base). At a separate locality (called McKay Head; locality in Supplemental information) ca. 2.5 km to the east, the basal McCoy Brook Formation consists of red mudstones resting directly on the NMB. A sample here from the basal 0.1 m produced an Ir concentration of 121 pg/g. The lowest Ir concentration in the McCoy Brook Formation, 11 pg/g, was measured in eolian sandstone 28 m above the formation base.

**Table 1:** Data summary. Mean values of analyses for Pd, Pt and Ir separated by peak and background Ir concentration. ( ) number of samples in mean.

Basin	Formation	Pd^a^ (ng/g)	Pt^a^ (ng/g)	Ir^b^ (pg/g)
Fundy	Scots Bay (base)	2.21 (2)	1.92 (2)	403 (2)
	Scots Bay (upper)			53 (4)
	McCoy Brook			27 (5)
	McCoy Brook (McKay Head)			121
Deerfield	FRB-3.0	1.62	0.72	90
	FRB other			36 (15)
	TF + 1.5			71
	TF other			38 (2)
Hartford	NHF(?) HCL (fine)	1.35 (2)	0.82 (2)	393 (4)
	NHF(?) HCL (coarse)			44 (2)
	NHF SR			36 (2)
Newark	PF TQ	2.29 (2)^c^	4.18 (2)^c^	645 (2)^c^

Scots Bay , Scots Bay Mbr of McCoy Brook Fm; FRB, Fall River beds of Sugarloaf Fm; − 3.0, 3 m below Deerfield Basalt; TF, Turners Falls Fm; + 1.5, 1.5 m above Deerfield Basalt top; NHF, New Haven Fm; HCL, Holyoke Clathropteris locality; SR, Silver Ridge; PF, Passaic Fm; TQ, Tilcon Quarry.

^a^Analysis by gravimetric lead fire assay.

^b^RNAA.

^c^Analysis by NSFA.

### Deerfield basin

The Deerfield basin is a northern extension of the Hartford basin, separated by a structural saddle, although the two basins are assumed to have been depositionally continuous^44^. The rift-fill sequence of the basin contains a single CAMP extrusive unit, the Deerfield Basalt (DB) considered correlative with the middle extrusive unit of CAMP and therefore Jurassic in age. The underlying sedimentary unit is the Sugarloaf Arkose, the uppermost eight meters of which, consisting of grayish–reddish mudstones, are informally named the Fall River Beds^44^. We sampled the Fall River Beds at 0.1 m spacing from the contact with the DB to a depth of 1.0 m below the DB, and continued sampling at 0.5 m spacing from 1.0 to 5.0 m below the DB (locations in supplemental data). The sedimentary unit above the DB is the (Lower Jurassic) Turners Falls Formation. We sampled the Turners Falls Formation at 0.5 m spacing from the contact with the underlying DB to a height of 2.0 m above the DB. All samples from the Deerfield basin yielded Ir levels < 100 pg/g (see Supplemental Table 2a), with the highest concentration, of 90 pg/g, more than double the mean value, measured 3.0 m below the DB base. A horizon containing abundant *Clathropteris* remains occurs at ca. 5 m below the DB, but no enrichment of Ir is found here. The sample from 3.0 m below the DB produced Pd and Pt concentrations of 1.6 and 0.7 ng/g, respectively. For strata above the DB, the highest Ir concentration for the sampled section was measured 1.5 m above the top of the DB. at 71 pg/g (Table 1). We consider this a possible, but at best marginal Ir anomaly. In general, the peak Ir values at this location are significantly lower than other Ir anomalies and we concede that we may have missed a more significant peak concentration due to the coarse sample intervals.

### Hartford basin

In the northern Hartford basin (near Holyoke, Massachusetts) there is an isolated, or “floating” outcrop that is not connected stratigraphically to any CAMP units^45^. The outcrop consists of ca. 9 m of mainly thick-bedded coarse-grained sandstone beds, but also contains a finer-grained interval that hosts a clay-rich layer of a few centimeters thickness. This bed contains abundant plant debris, particularly whole specimens of *Clathropteris*, and trilete spores. Although by no means certain, the lithology suggests that the outcrop belongs to the New Haven Formation, and palynology of the fine-grained interval suggests a potential correlation to the “fern spike” locality of the Jacksonwald syncline^30,31,45^. Four samples of the fine-grained, *Clathropteris*-bearing layer yielded Ir concentrations > 200 pg/g, to a maximum level of 542 pg/g. Pd and Pt concentrations from this layer up to 1.8 ng/g and 0.9 ng/g, respectively (Table 1 and Supplemental Table 3a). Samples from slightly coarser-grained strata immediately above and below, but lacking plant debris, produced Ir values an order of magnitude lower. A separate locality in the southern Hartford basin (at Silver Ridge, Connecticut) exposes a bed bearing abundant plant remains in the uppermost New Haven Formation below the Talcott Basalt (TC), the basal CAMP flow in the Hartford basin^45^. This horizon also is potentially correlative with the “fern-spike” horizon in the Newark basin, but analyses of samples from this layer showed no elevation of Ir.

### Newark basin

The original Ir enrichment in the Newark Supergroup was found in the upper Passaic Formation in the Jacksonwald syncline of the westernmost Newark basin^30,31^. We sampled a potentially correlative horizon in the uppermost Passaic Formation, which underlies the OMB ca. 150 km to the northeast of the original location (location in Supplemental information) at the (now abandoned) Tilcon quarry in the northern Newark basin (location in Supplemental Table 4). The uppermost Passaic Formation here contains fine-grained layers with abundant carbonized plant remains including *Clathropteris*^46^. Samples of a carbonaceous, plant-rich mudstone horizon ca. 1.8 m below the contact with the OMB yielded Ir concentrations up to 780 pg/g, Pd to a maximum of 2.8 ng/g and Pt up to 5.4 ng/g (Table 1). Despite the differences in thicknesses of the overlying Passaic Formation section, this horizon potentially correlates with the fern spike horizon in the western Newark basin.

## Discussion

We document multiple occurrences of Ir enrichment above background levels, and above typical levels for continental crust (ca. 40 pg/g^47^) in terrestrial sediments surrounding the TJB. The CAMP flows in the Newark Supergroup basins provide a framework of reference for the age of the Ir-enriched horizons through interbasinal correlation of the three distinct flow units (Fig. 2). The highest levels of Ir enrichment found in this study are in the uppermost Passaic Formation (Tilcon Quarry) and in strata presumed to represent the upper New Haven Formation in the northern Hartford basin (Holyoke *Clathropteris* locality). These new data, combined with previous data from the Fundy and western Newark basins^30,31,35,36^, suggest the potential presence of a widespread, correlative, Ir-enriched horizon below the base of the oldest CAMP flow in the Newark basins. All of the horizons of elevated Ir that lie below the oldest CAMP flows share similar palynological assemblages, the so-called fern spikes, and common macrofloral elements^8,48,49^. However, horizons in the Deerfield basin, in the basal Fall River Beds (5.0 m below the DB), and in the Hartford basin at the Silver Ridge locality, that appear to correlate palynologically with this horizon showed no Ir enrichment. There are multiple possible explanations for the discrepancy: (1) there in fact is no single widespread, correlative Ir-enriched horizon below the lowest CAMP unit because the observed enrichments are strictly local phenomena; (2) a correlative horizon exists, but the horizons sampled in the Deerfield basin (5.0 below the DB) and Hartford basin (below the TB at Silver Ridge) are not correlative stratigraphically with the horizons of enrichment in the Fundy and Newark basins as thought; or (3) the enriched horizons were missed during sampling due to a coarse sample spacing. Data are insufficient at this time to resolve amongst these hypotheses.

The very modest Ir enrichment (90 pg/g) seen in the Fall River Beds at 3.0 m below the DB potentially may correlate temporally with the oldest CAMP flows in North America (OMB in Newark, NMB in Fundy and TC in Hartford). This horizon lies ca. 2 m higher than the horizon previously identified as the TJB^44^. Using the modern definition of the TJB^8^ (Fig. 2), we tentatively place the TJB above the zone of modest Ir enrichment in the Fall River Beds (i.e. less than 3.0 m below the DB). Similarly, the very modest Ir enrichment (71 pg/g) 1.5 m above the DB may mark the third eruptive phase of the Newark CAMP units (the Hampden and Hook Mountain basalts of the Hartford and Newark basins, respectively). Although the Scots Bay strata overlie the NMB at an angle, the basal Scots Bay beds are identified palynologically as uppermost Triassic^8^. Therefore, there is very little temporal separation between the deposition of the basal Scots Bay strata and the termination of the eruption the NMB and we do not correlate the Scots Bay high-Ir zone with later CAMP eruptive episodes.

**Figure 3:**
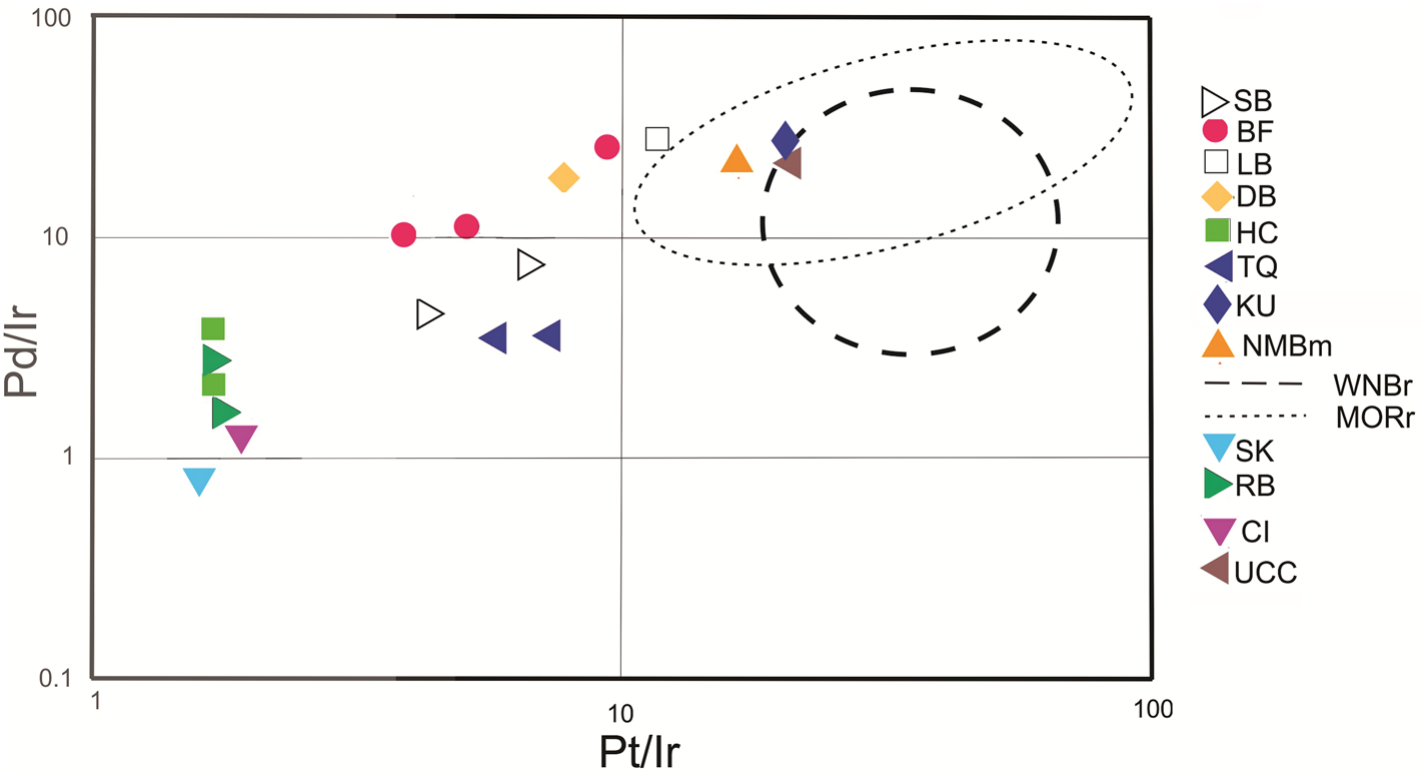
PGE ratio crossplot. Pt/Ir–Pd/Ir crossplot for Newark Supergoup samples compared to CAMP volcanics and intrusives, CI chondrite, K-Pg boundary clays, average crust and mantle. SB, Scots Bay Member (McCoy Brook Formation) samples SB-1, SB-2; BF, Blomidon Formation, Ir peak samples PI-10, PI-30, PI-45^35^; LB, Blomidon Formation as above, but mean of samples PI-50-60^35^ in lower part of sample interval with Ir at or below average continental crust; DB, Deerfield Basin sample DB-3.0; HC, Holyoke *Clathropteris* locality (New Haven Formation) samples HC-F-1, HC-F-2; TQ, Tilcon Quarry (Passaic Formation) samples TQ-1, TQ-2; NMBm, North Mountain Basalt mean ratios^51^; WNBr, approximate range of ratios for western Newark basin intrusions^50^; MORr, approximate range of ratios for Moroccan CAMP units^53^; SK, Stevns Klint K-Pg layer^54^; RB, Raton Basin^54^; CI, CI chondrite^54^; UCC, mean upper continental crust ratio^73^.

The geochemical composition of the zones of Ir enrichment, in particular, the PGE ratios, may provide evidence of the source of the Ir, i.e., magmatic or extraterrestrial. Geochemical data from intrusions (dikes and sills) in the western Newark basin associated with the CAMP extrusions^50^ provide a useful comparison to the PGE data from sedimentary strata of the Newark Supergroup. Although the analytical resolution of the instruments in use at that time was coarse (the Ir detection limit was ca. 500 pg/g), the data are broadly useful. The maximum Ir concentration measured was 2600 pg/g and the mean Ir (for 66 samples representing 10 locations) was ca. 600 pg/g, yielding mean Pd/Ir and Pt/Ir ratios of 14.8 and 22.2, respectively. An extensive geochemical data set also exists for the NMB in the Fundy basin^51^. The NMB has an internal stratigraphy comprising three flow units^52^. The work by Greenough and Fryer^51^ documented that the lowest unit is Ir-rich in comparison to other units, particularly in orthopyroxene-enriched basalts, with concentrations up to 1200 pg/g and a mean of 252 pg/g for the lower flow unit (N = 20). The mean concentrations of Pt and Pd are more than an order of magnitude greater at 4146 pg/g and 6254 pg/g, respectively. The mean Pt/Ir (= 16) and Pd/Ir (= 24.8) ratios derived from these data accord well with the data from the western Newark basin intrusives^50^ (Fig. 3) as well as recently published data for Moroccan CAMP volcanics^53^.

None of the samples from peak-Ir horizons identified in this study have Pt/Ir and Pd/Ir ratios that match those in CAMP flows and intrusives, although the Pd/Ir ratios of samples from the Fundy and Deerfield basins overlap typical CAMP values. Pt/Ir ratios of the samples are lower than CAMP in all samples. Specifically, samples from the Blomidon Formation^35^ (below the NMB) and the Scots Bay Member (above the NMB) in the Fundy basin, as well as the very small enrichment found in the Fall River beds (below the DB) in the Deerfield basin, have Pd/Ir ratios (5.8–18.0) that are similar to those for CAMP flows and intrusives, but the Pt/Ir ratios (3.8–9.5) are comparatively lower (Fig. 3). The Pd/Ir ratios for the Newark basin samples (3.5–3.6) are slightly lower than those for the Deerfield and Fundy basins, although the Pt/Ir ratios (5.8–6.9) are similar. In contrast to the other samples, the samples from the New Haven Formation (Holyoke *Clathropteris* locality) in the Hartford basin yield very low Pd/Ir (2.1–3.4) and Pt/Ir (1.7) ratios. These values are closer to the PGE ratios for CI chondrite as well as those for K-Pg impact beds in both terrestrial and marine environments^54^. Notably, the Hartford basin (*Clathropteris* locality) samples, which have significant Ir anomalies, have much lower Pd and Pt concentrations than those in the Fundy and Newark basins.

For comparison to a section from the marine realm, we note here that analysis of PGEs across a deep marine chert-shale TJB section in Japan^39^ yielded maximum Ir values of 219 pg/g and above the boundary Ir was measured at a maximum of 131 pg/g. The authors noted that the Pd/Pt and Ir/Pt ratios and chondrite-normalized PGE profile all show close affinity to previous analyses of CAMP volcanics and do not resemble the PGE ratios of the K-Pg boundary clays.

**Figure 4:**
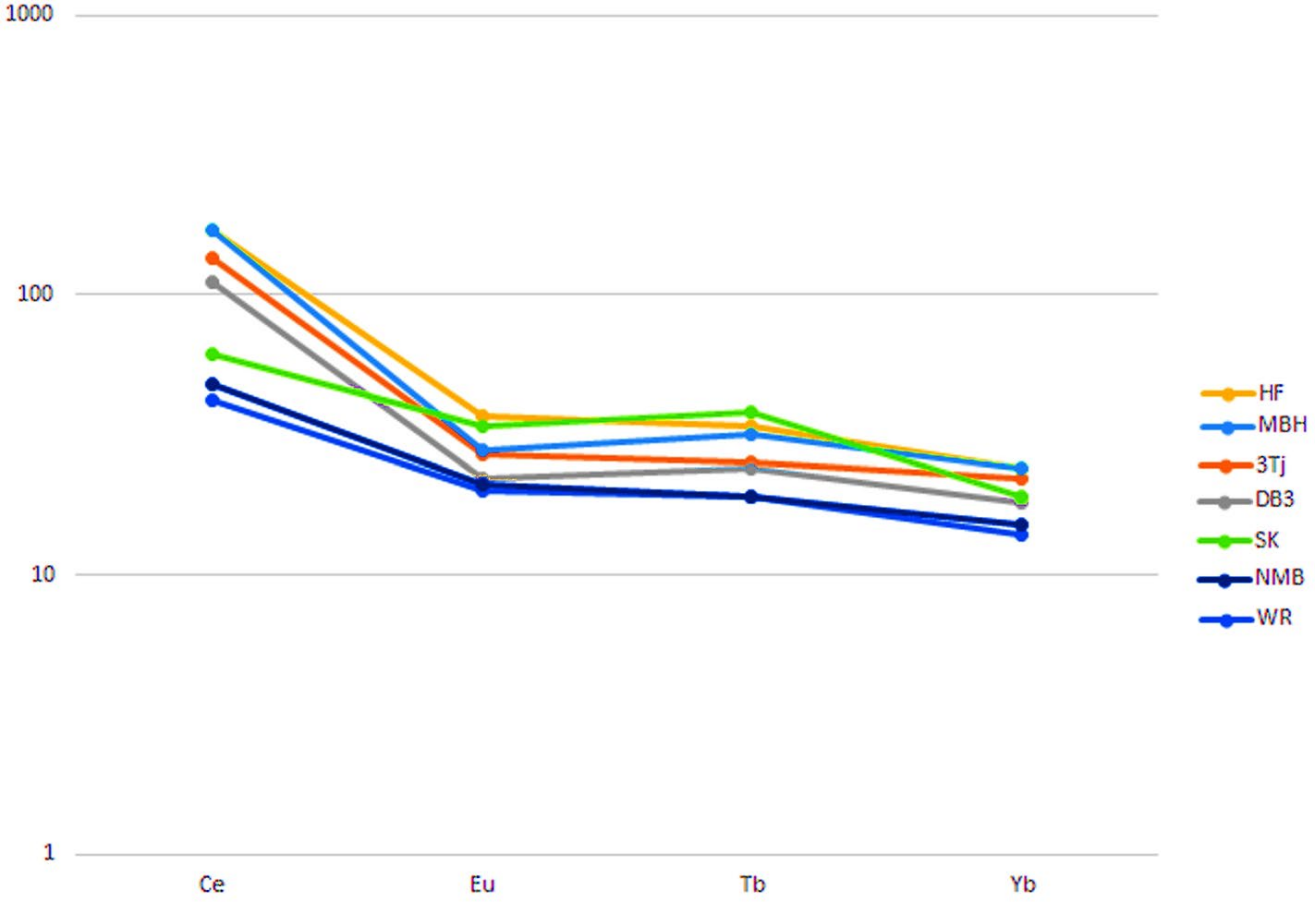
REE profiles for Newark Supergroup. Profiles for selected chondrite normalized REEs from Ir-enriched horizons compared to CAMP units: HF, Holyoke *Clathropteris* locality of New Haven Formation, fine-grained facies; MBH, McKay Head locality of basal McCoy Brook Formation; 3Tj = Jacksonwald Syncline location Passaic Formation (data from^30,31^); DB3, Deerfield basin Fall River Beds at 3 m below DB; SK, data for Stevns Klint Fish Bed^53^; NMB, North Mountain Basalt^51^; WR, West Rock sheet of western Newark basin intrusions^50^.

Trace element analyses of selected samples from the Fundy, Deerfield and Hartford basins (Supplemental Tables 1b, 2b, 3b) include a subset of four REEs (Ce, Eu, Tb and Yb). Chondrite-normalized profiles for these samples match closely (Fig. 4) and also match a profile constructed from the Jacksonwald Syncline data (in the supplemental information)^30^. These profiles parallel those shown for the NMB and Newark basin intrusives, as well as a profile for the K-Pg boundary^55^, with the exception of a slight Ce elevation. Likely, these profiles reflect REE input partially or even primarily from continental sediments rather than volcanic input^56^.

The large variation in Pt/Ir and Pd/Ir ratios in these data suggest that the conditions of PGE enrichment are not uniform across the basins. Whether these variations reflect variable sources or local modification, or both, remains subject to speculation. Variations in sedimentation rates alone are capable of causing Ir enrichment in deep marine environments by concentration of the cosmic micrometeorite flux during slow sedimentation^57^, but this mechanism is not applicable in alluvial terrestrial depositional environments due to their inherently dynamic nature. Therefore, we examine alternative mechanisms of enrichment in sediments. As the impact hypothesis for the Ir enrichment^30^ is no longer considered viable^36,53^, the CAMP volcanics are the remaining logical source for Ir and other PGEs in sediments surrounding the TJB at abundances beyond typical crustal levels. The presence of anomalous concentrations of Hg also has been observed in TJB sediments in both terrestrial and marine sediments^29^, including the multiple horizons at the same stratigraphic level in the uppermost Blomidon Formation where Ir enrichment is documented^35,36^, supporting a volcanic source for the Ir.

The elevated PGEs in the upper meter of the Blomidon Formation were interpreted as having been derived directly from the overlying NMB by leaching associated with post-eruption hydrothermal activity^35^, such as has been documented in the Scots Bay Member^37^. We presently consider leaching of PGEs directly from CAMP basalts by hydrothermal activity as a viable mechanism for releasing PGEs from the CAMP basalts and remobilizing them in the surrounding formations. Specifically, we cite the Fundy basin, including the Blomidon Formation below the NMB and the Scots Bay Member above the NMB. Evidence for leaching of heavy metals from the basalt flows in other basins includes numerous abandoned copper mines in the Mesozoic sediments and basalts of the Newark basin^58,59^. The ore zones of most of these mines occur in bleached gray "hornfels" 2.0 m or less below the OMB, in gray or black organic enriched layers 4 m or less below the OMB, or within 2 m of Palisades-related intrusions. Notably, thin (ca. 2 mm) green lenses of chrysocolla, or less commonly malachite, occur in the layer sampled for PGE content at the Tilcon Quarry.

The higher concentrations of Ir in the Blomidon Formation (Fundy basin) were generally found in multiple drab-colored horizons containing organic-rich laminae in an otherwise oxidized red mudstone formation^35^. Subsequent detailed analyses found a positive correlation between Ir and total organic carbon^36^ (TOC) leading to the suggestion that Ir was mobile in the sedimentary column and sequestration was controlled by the presence of organic matter and the resultant redox boundaries, a possibility suggested previously^60^. However, analysis of the marine section in the Eiberg basin (the Rhaetian-Hettangian GSSP section at Kuhjoch) demonstrated a lack of covariance of Ir with total organic content^40^. Nonetheless, Ir was found to covary with the elements V and Zn, both considered indicators of reducing conditions, although true dysaerobic conditions in the section were apparent only in a single bed. Moreover, it has been suggested that redox conditions are not as important in concentrating Ir as the presence of organic compounds which form organometallic complexes with Ir^61^, and coincidentally, also with Hg^62^.

Although Ir has been considered particularly immobile among the PGEs^54^, Ir and other PGEs have exhibited varying mobility in marine sediments. For example, one study of a K-Pg boundary core of abyssal clays^63^ found only slow diffusion of Ir within the sedimentary column without significant fractionation among the PGEs. Yet, a different study noted that Pt/Ir and Pd/Ir ratios varied by a factor of two across a few cm of the K-Pg boundaries in Denmark and Spain^64^. Importantly, the mobility of Ir in terrestrial environments has been documented in response to conditions of high acidity^65^. In our study, we noted high Ir, Pd and Pt concentrations at horizons containing abundant plant remains in the Hartford basin (Holyoke *Clathropteris* locality), the northern Newark basin (Tilcon Quarry) and the original sample locality in the western Newark basin^30,31^. Acidification of terrestrial surface environments due to the formation of aerosols from outgassed SO_2_, and to a lesser extent dissolution of CO_2_ in meteoric waters, as well as fluorides and chlorides released during the CAMP eruptions have long been thought to have contributed to the floral turnover at the TJB^2,4,66,67^. Potentially, the concentrations of plant matter below the oldest CAMP flows in North America record widespread plant mortality in response to the initial phase of the eruptions. As noted earlier, some studies have suggested that the initiation of the CAMP eruptions was earlier in Morocco than in North America^12,13^; this may have caused heightened plant mortality through acid deposition and heavy metal toxicity^16,18,19^, as well as providing an initial source of Ir through outgassing. Subsequent releases of Ir and associated PGEs may have been subject to pH-enhanced mobilization in the sedimentary column, with sequestration focused by formation of organometallic complexes at specific organic-rich horizons, including those recording eruption-related plant mortality.

It is unclear how much stratigraphic separation between the source and the horizon of enrichment can accommodate mobilization of PGEs by leaching or acidification of the surface environment; the Tilcon Quarry horizon occurs ca. 1.8 m below the OMB, the Fall River Bed horizon is 3 m below the DB and the western Newark basin horizon^30,31^ is ca. 15 m below the OMB. Furthermore, there is no overlying Talcott Basalt at the Holyoke *Clathropteris* locality, although the Holyoke Basalt would have been present but higher stratigraphically. Consequently, we suggest that elevated Ir in the TJB sediments likely reflects mechanisms of concentration in addition to leaching, specifically, by atmospheric fallout. For example, high levels of Ir have been noted in the plumes of basalt eruptions, particularly in ocean-island basalts^68–70^. Despite a high level of uncertainty, the mass-balance calculations indicate that the volcanic plume hypothesis is a plausible explanation for the global distribution of Ir at the levels found in the Fundy basin^36^. We suggest that elevated Ir concentrations at some locations, such as above and below the DB in the Deerfield basin and the Holyoke *Clathropteris* locality may have been sourced from CAMP aerosols and possibly ash in volcanic plumes, rather than leaching of basalt. Similarly, elevated Ir concentrations in marine TJB sections^38–40^ undoubtedly resulted from fallout from volcanic plumes during the CAMP eruptions. Nevertheless, leaching may be the more likely source of enrichment where proximity to the CAMP flows and evidence of post-eruptive hydrothermal activity exists, as observed in the Fundy and Newark basins.

Regardless of the source (leaching or plume deposition) mobilization in the sedimentary column may have been accompanied by fractionation among the PGEs. In the Blomidon Formation, where multiple horizons of enrichment occur, Pd/Ir and Pt/Ir vary throughout the sample interval, with more Ir-enriched horizons typically displaying lower ratios and low-Ir horizons exhibiting ratios closer to those of the CAMP magmatics^35^ (Fig. 3). The weak covariance of Ir with both Pd and Pt^35^ suggests either differential mobility in the sedimentary column or differential response of Ir, Pd and Pt to the presence of organic matter and/or redox boundaries. Fractionation during mobilization may best explain the Pd/Ir and Pt/Ir ratios at other locations, e.g., the Newark basin (Tilcon Quarry) and the more extreme case in the Hartford basin (Holyoke *Clathropteris* locality), where fractionation resulted in Pd/Ir and Pt/Ir ratios approaching chondritic values. Conversely, the close affinity of the deep marine section in Japan^39^ with typical CAMP ratios suggests immobility and lack of PGE ratio modification in this particular setting.

In conclusion, we suggest that horizons of Ir and associated PGE enrichment are widespread across the Newark Group basins both below and within the CAMP extrusive zone and may reflect derivation of these metals from multiple mechanisms. In particular, we find evidence for hydrothermal activity that may have leached PGEs directly from the CAMP volcanics, but also acknowledge the potential origin from the fallout of aerosols and/or ash. Thus, elevated Ir may have resulted from strictly local processes in proximity to large flood basalt provinces, whereas only an airborne mechanism can explain anomalies of Ir and other PGEs in sedimentary sections located distal to large flood basalts and in marine sections not subject to major changes in depositional rate. Notably, both mechanisms are compatible with the mobilization of PGEs, with fractionation and enrichment in horizons where organic matter allowed formation of organometallic complexes. This conclusion implies that PGE ratios may not be diagnostic in identifying the origin of the PGEs as these ratios may vary from an affinity to a magmatic source to one of an extraterrestrial (e.g. chondritic) source.

## Methods

Iridium was analyzed in all samples from the Scots Bay Member, Deerfield basin and Hartford basin by radiochemical neutron activation analysis (RNAA). A one-gram split of each sample was heated overnight at 105 °C and then ground to a powder in a high-purity alumina mortar in a clean room. A ~ 200 mg split from each sample was sealed in a quartz-glass vial and irradiated for 30 h in the University of Missouri Research Reactor at a neutron flux of 5 × 10^13^ n cm^–2^ s^−1^. Three months following the irradiation, Ir was chemically purified using a method similar to that described by Schellenberg et al.^71^. Samples were counted on coaxial intrinsic Ge gamma-ray detectors (with resolution from 1.75 to 1.90 keV at 1.3 meV) for 24–48 h. Typical 1-sigma counting errors for Ir are 5–20 relative percent, except for very low concentrations below 10 pg/g for which errors could be 30–40%. Results of Ir RNAA are compiled in the Supplemental information set. Selected samples from the RNAA set were analyzed for trace element concentrations by instrumental neutron activation analysis (INAA). Prior to the RNAA chemistry, the sample powders were counted for ca. 3 h to determine concentrations of Sc, Cr, Fe, Co, Ni, Zn, Rb, Cs, Ce, Eu, Tb, Yb, Hf, Ta and Th. Complete results of INAA are also compiled in the Supplemental information set.

For the samples with elevated Ir identified by RNAA as described above a 20-g split from the original sample was analyzed by gravimetric lead fire assay technique with ICP-MS measurement to determine the concentrations of Au, Pd and Pt. These analyses were conducted by Geoscience Laboratories (Sudbury, Ontario), a division of the Ontario Geological Survey^72^. Only the Tilcon Quarry samples were analyzed for Au, Pd, Pt, Ir, Rh and Ru by nickel-sulfide fire assay with ICP-MS finish in which samples were fused with sodium carbonate in the presence of Ni and S to produce a nickel sulfide button that subsequently was digested by concentrated HCl. The metals were then co-precipitated with Te and redissolved in aqua regia for analysis by a Perkins-Elmer ELAN 5000 inductively coupled plasma mass spectrometer. The detection limits, calibrated to internal standards, are as follows: Ir = 0.03 ng/g; Au = 0.27 ng/g; Pd = 0.06 ng/g; Pt = 0.10 ng/g; Rh = 0.03 ng/g; and Ru = 0.09 ng/g. Replicate analyses indicate a measurement precision of ± 0.02 ng/g.

## Supplementary information

Supplementary Information.

## Data Availability

The data shown and discussed in this paper are presented in full in the Supplementary information.
