# Supplementary material for: Widespread elevated iridium in Upper Triassic–Lower Jurassic strata of the Newark Supergroup: implications for use as an extinction marker
Source: Sci Rep. 2020 Nov 11;10:19575. doi: 10.1038/s41598-020-76238-4 (PMC7659020; doi:10.1038/s41598-020-76238-4)
Supplement: Supplementary file 1 — Supplementary Information. [file 41598_2020_76238_MOESM1_ESM.pdf]

Supplemental Information for

Widespread elevated iridium in Upper Triassic-Lower Jurassic strata of the Newark Supergroup: Implications for use as an extinction marker

Lawrence H. Tanner[1] ✉, Frank T. Kyte[2], John H. Puffer[3]

[1]Department of Biological and Environmental Sciences, Le Moyne College, Syracuse, NY 13214 USA. [2]Institute of Geophysics and Planetary Physics, University of California, Los Angeles, CA 90095-1567 USA. [3]Department of Earth and Environmental Science, Rutgers University, Newark New Jersey 07102 USA. ✉email: tannerlh@lemoyne.edu

Supplemental Table 1a. Fundy Basin LFA and RNAA data.

Formation	Member/lithology	ID	Strat position	Au ng/g (LFA)	Ir pg/g (RNAA)	Pd ng/g (LFA)	Pt ng/g (LFA)
N 45º 23.605', W 64º 14.292'							
McCoy Brook	Scots Bay	SB-1	NMB + 0.04 m	6.3	247	2.03	1.55
McCoy Brook	Scots Bay	SB-2	NMB + 0.05 m	9.3	559	2.38	2.29
McCoy Brook	Scots Bay	FB-3	NMB + 0.10 m		74		
McCoy Brook	Scots Bay	FB-4	NMB + 0.20 m		56		
McCoy Brook	Eolian	MBE	NMB + 28 m		11		
N 45º 23.224', W 64º 15.036'							
McCoy Brook	upper lake beds	MBL-1	NMB + 30.10 m		25		
McCoy Brook	upper lake beds	MBL-2	NMB + 30.20 m		21		
McCoy Brook	upper lake beds	MBL-3	NMB + 30.30 m		33		
McCoy Brook	upper lake beds	MBL-4	NMB + 30.40 m		43		
N 45º 23.838', W 64º 12.199'							
McCoy Brook	McKay Head	MBH	NMB + 0.10 m		121		

NMB = North Mountain Basalt

Supplemental Table 1b. INAA of Fundy Basin samples in supplemental table 1a.

Formation	Lithology	ID	Strat position	Sc µg/g	Cr µg/g	Fe mg/g	Co µg/g	Ni µg/g	Zn µg/g	Rb µg/g	Cs µg/g	Ce µg/g	Eu µg/g	Tb µg/g	Yb µg/g	Hf µg/g	Ta µg/g	Th µg/g
McCoy Brook	MB Eolian	MBE	NMB + 28 m	1.9	10.3	5.3	2.1	70.6	10.3	30.3	1.1	37.5	0.6	0.3	0.9	5.7	0.4	3.4
McCoy Brook	upper lake beds	MBL-1	NMB + 30.10 m	14.4	81.7	41.2	13.9	118.6	82.4	195.2	21.4	80.4	1.3	0.8	2.2	4.8	0.9	10.8
McCoy Brook	upper lake beds	MBL-2	NMB + 30.20 m	14.0	78.7	36.9	13.4	62.1	75.4	179.8	19.8	71.7	1.2	0.8	2.3	3.9	1.0	10.1
McCoy Brook	upper lake beds	MBL-3	NMB + 30.30 m	11.7	66.1	33.8	11.2	23.8	41.4	140.8	14.7	141.5	2.1	1.2	2.7	4.9	0.8	7.9
McCoy Brook	upper lake beds	MBL-4	NMB + 30.40 m	7.7	37.5	24.0	7.0	7.0	38.7	80.3	7.4	219.4	3.1	1.7	2.8	3.8	0.8	5.4
McCoy Brook	McKay Head	MBH	NMB + 0.10 m	12.5	33.8	21.1	19.8	<87.83	36.7	68.1	2.6	107.1	1.6	1.2	3.8	3.6	0.7	13.3

Supplemental Table 2a. Deerfield Basin LFA and RNAA data.

N 42º 37.005', W 72º 33.166' below DB						
Formation	Member	Strat position	Au ng/g (LFA)	Ir pg/g (RNAA)	Pd ng/g (LFA)	Pt ng/g (LFA)
Sugarloaf Fm	Fall River Beds	DB-5.0		16		
Sugarloaf Fm	Fall River Beds	DB-4.0		28		
Sugarloaf Fm	Fall River Beds	DB-3.0	7.4	90	1.62	0.72
Sugarloaf Fm	Fall River Beds	DB-2.5		54		
Sugarloaf Fm	Fall River Beds	DB-2.0		37		
Sugarloaf Fm	Fall River Beds	DB-1.5		31		
Sugarloaf Fm	Fall River Beds	DB-1.0		38		
Sugarloaf Fm	Fall River Beds	DB-0.9		36		
Sugarloaf Fm	Fall River Beds	DB-0.8		37		
Sugarloaf Fm	Fall River Beds	DB-0.7		35		
Sugarloaf Fm	Fall River Beds	DB-0.6		30		
Sugarloaf Fm	Fall River Beds	DB-0.5		31		
Sugarloaf Fm	Fall River Beds	DB-0.4		36		
Sugarloaf Fm	Fall River Beds	DB-0.3		44		
Sugarloaf Fm	Fall River Beds	DB-0.2		49		
Sugarloaf Fm	Fall River Beds	DB-0.1		34		
N 42º 36.879', W 72º 33.084' above DB						
Turners Falls Fm		DB+0.1		31		
Turners Falls Fm		DB+0.5		44		
Turners Falls Fm		DB+1.5		71		
Turners Falls Fm		DB+2.0		40		

DB = Deerfield Basalt

Supplemental Table 2b. Deerfield Basin INAA of samples in supplemental table 2a.

Formation	Member	Strat position	Sc µg/g	Cr µg/g	Fe mg/g	Co µg/g	Ni µg/g	Zn µg/g	Rb µg/g	Cs µg/g	Ce µg/g	Eu µg/g	Tb µg/g	Yb µg/g	Hf µg/g	Ta µg/g	Th µg/g
Sugarloaf Fm	Fall River Beds	DB-5.0	26.3	126	48.0	18	47.1	87.0	180	7.7	114.8	1.5	1.2	3.6	8.3	1.3	14.8
Sugarloaf Fm	Fall River Beds	DB-4.0	14.7	91	48.4	18	59.1	94.6	88	3.5	97.4	1.5	1.1	3.2	6.2	1.6	14.3
Sugarloaf Fm	Fall River Beds	DB-3.0	24.5	112	46.5	22	58.9	96.0	199	12.2	69.3	1.2	0.9	2.9	6.5	1.2	13.0
Sugarloaf Fm	Fall River Beds	DB-2.5	16.0	72	33.6	13	39.5	49.4	115	6.0	88.9	1.8	1.2	3.7	6.3	1.5	11.4
Sugarloaf Fm	Fall River Beds	DB-2.0	15.6	73	37.0	13	25.8	69.7	111	4.6	127.9	1.8	1.5	3.8	8.7	1.2	17.4
Sugarloaf Fm	Fall River Beds	DB-1.5	18.3	88	38.4	22	76.2	73.0	163	8.9	85.1	1.6	1.3	3.5	9.4	1.3	14.5
Sugarloaf Fm	Fall River Beds	DB-1.0	18.6	83	43.7	18	28.1	102.2	169	10.4	97.8	2.2	1.6	4.1	7.9	1.2	13.7
Sugarloaf Fm	Fall River Beds	DB-0.9	18.3	88	48.7	17	42.5	64.0	188	10.9	80.3	1.5	1.1	3.3	7.5	1.2	11.8
Sugarloaf Fm	Fall River Beds	DB-0.8	18.1	88	49.3	19	58.8	66.4	203	13.5	67.9	1.2	0.9	2.8	7.1	1.4	11.7
Sugarloaf Fm	Fall River Beds	DB-0.7	17.2	74	31.7	11	19.4	43.8	104	5.1	107.1	2.1	1.4	3.7	6.9	1.0	14.3
Sugarloaf Fm	Fall River Beds	DB-0.6	12.6	63	28.9	10	45.0	38.9	99	4.3	111.0	1.8	1.2	3.3	7.7	0.9	13.6
Sugarloaf Fm	Fall River Beds	DB-0.5	15.9	74	43.8	13	25.2	59.0	151	6.0	67.8	1.3	0.9	3.1	8.1	1.2	11.8
Sugarloaf Fm	Fall River Beds	DB-0.4	13.1	70	37.8	15	43.0	50.6	86	3.3	74.4	1.4	0.9	2.7	6.7	1.0	12.1
Sugarloaf Fm	Fall River Beds	DB-0.3	19.7	104	60.6	22	19.2	79.0	202	12.4	95.9	1.8	1.3	3.8	9.5	1.4	16.0
Sugarloaf Fm	Fall River Beds	DB-0.2	16.3	81	57.2	23	50.9	79.0	189	14.2	109.6	1.9	1.3	3.6	7.6	1.1	13.5
Sugarloaf Fm	Fall River Beds	DB-0.1	15.1	73	40.2	22	57.9	70.3	124	9.1	96.1	1.5	1.0	3.1	8.0	1.0	12.7
Turners Falls Fm		DB+0.1	10.7	56	49.1	29	54.2	93.2	55	4.8	57.2	1.4	1.0	2.8	6.8	1.0	9.9
Turners Falls Fm		DB+0.5	15.5	95	51.4	16	40.9	62.6	140	9.0	96.1	1.5	1.0	3.0	5.9	1.2	13.5
Turners Falls Fm		DB+1.5	25.5	93	52.5	20	<47	60.5	186	12.4	147.6	1.9	1.5	3.5	8.6	1.6	21.8
Turners Falls Fm		DB+2.0	12.8	60	28.5	11	65.5	38.6	125	8.5	68.4	1.7	0.9	2.6	4.5	0.9	7.8

Supplemental Table 3a. Hartford Basin LFA and RNAA data.

Formation	ID	lithology	Strat position	Au ng/g (LFA)	Ir pg/g (RNAA)	Pd ng/g (LFA)	Pt ng/g (LFA)
N 42° 13.018', W 72° 39.781' Holyoke Clathropteris locality							
New Haven Fm (presumed)	HC-F-1	fine-grained	0.01 m from base	15.1	542	1.83	0.94
New Haven Fm	HC-F-2	fine-grained	0.02 m	8	409	0.86	0.7
New Haven Fm	HC-F-3	fine-grained	0.03 m		386		
New Haven Fm	HC-F-4	fine-grained	0.04 m		244		
New Haven Fm	HC-C-1	coarse-grained	0.1 m above fine layer		52		
New Haven Fm	HC-C-2	coarse-grained	0.1 m below fine layer		36		
N 41° 35.178', W 72° 45.688' Silver Ridge							
New Haven Fm	HB-3		TB - 1.5 m		51		
New Haven Fm	HB-4		TB - 1.6 m		61		

TB = Talcott Basalt

Supplemental Table 3b. Hartford Basin, Holyoke *Clathropteris* locality INAA for samples in supplemental table 3a.

Formation	ID	lithology	Strat position	Sc µg/g	Cr µg/g	Fe mg/g	Co µg/g	Ni µg/g	Zn µg/g	Rb µg/g	Cs µg/g	Ce µg/g	Eu µg/g	Tb µg/g	Yb µg/g	Hf µg/g	Ta µg/g	Th µg/g
New Haven	HC-F-1	fine-grained	0.01 m	17.9	101.9	97.1	80.4	86.1	97.3	211.3	2.8	111.9	2.2	1.3	4.5	3.0	1.5	13.7
New Haven	HC-F-2	fine-grained	0.02 m	13.5	104.3	51.0	10.0	<82.83	65.2	204.6	2.1	98.6	2.0	1.2	3.1	4.7	1.6	20.9
New Haven	HC-C-1	coarse-grained	above fine	20.3	99.0	50.1	6.2	80.6	67.3	115.2	3.1	92.0	1.1	0.9	2.5	6.1	1.3	15.0
New Haven	HC-C-2	coarse-grained	below fine	21.5	107.3	54.2	7.8	<116.6	66.0	123.1	3.0	88.2	1.1	0.8	2.5	6.6	1.3	14.6

Supplemental Table 4. Newark Basin NSFA data.

N 40° 52.750', W 74° 11.311’ Tilcon Quarry

Formation	ID	Strat poition	Au ng/g	Ir pg/g	Pd ng/g	Pt ng/g	Rh ng/g	Ru ng/g
Passaic Fm	TQ-1	OMB - 1.80 m	9	780	2.77	5.38	0.43	1.47
Passaic Fm	TQ-2	OMB - 1.82 m	4.3	510	1.81	2.98	0.29	0.92

TQ = Tilcon Quarry, OMB = Orange Mountain Basalt
